# Erratum to “Neurocognitive reorganization between crystallized intelligence, fluid intelligence and white matter microstructure in two age-heterogeneous developmental cohorts” [Dev. Cogn. Neurosci. 41 (2020) 100743]

**DOI:** 10.1016/j.dcn.2020.100769

**Published:** 2020-02-12

**Authors:** Ivan L. Simpson-Kent, Delia Fuhrmann, Joe Bathelt, Jascha Achterberg, Gesa Sophia Borgeest, Rogier A. Kievit, the CALM Team

**Affiliations:** a MRC Cognition and Brain Sciences Unit, University of Cambridge, Cambridge, Cambridgeshire, CB2 7EF, UK; b Dutch Autism & ADHD Research Center, Brain & Cognition, University of Amsterdam, 1018 WS Amsterdam, the Netherlands

The publisher regrets that the order of authors was incorrect at the time the article was published. This has now been corrected.

The publisher would like to apologise for any inconvenience caused.

