# Supplementary material for: Neurocognitive reorganization between crystallized intelligence, fluid intelligence and white matter microstructure in two age-heterogeneous developmental cohorts
Source: Dev Cogn Neurosci. 2019 Dec 9;41:100743. doi: 10.1016/j.dcn.2019.100743 (PMC6983934; doi:10.1016/j.dcn.2019.100743)
Supplement: Supplementary file 1 [file mmc1.docx]

**Supplementary Material**

**Is the Peabody Picture Vocabulary Test a measure of fluid ability?**

As a non-preregistered exploratory analysis, we more closely examined the cross-loading of the Peabody Picture Vocabulary Test (PPVT). This task asks participants to select the correct picture (out of four multiple-choice options) corresponding to the meaning of a word spoken by an examiner (Dunn and Dunn, 2007). As discussed previously in the Results section 3.1, modification indices suggested the PPVT should either be cross-loaded or solely loaded onto gf. To better understand this cross-loading, we performed an exploratory (i.e. not part of preregistration) analysis using SEM tree analysis. In this analysis, we allowed the PPVT to load on both gc and gf, and examined whether using age as a covariate yielded a developmental period where the associations between the latent factors and the PPVT task differed. This generated an age split for gf at around age 9.5 whereby the loading of the PPVT decreased (from 1 to .87, unstandardized estimate).

Conversely, for gc the loading remained the same (.12, unstandardized estimate). This suggested the PPVT as commonly implemented behaved as a fluid, rather than a crystallized, task, especially in younger participants of lower ability. Although purportedly a test of crystallized knowledge, the implementation of the PPVT may very well rely on more fluid, executive components including response selection and reasoning, especially in a cohort of children and adolescents with comparatively low overall performance.

A likely explanation for this pattern is that, while PPVT draws on gc, the demanding nature of the task may require more fluid, executive components in younger children, especially in a cohort with comparatively low overall performance (e.g. CALM). Moreover, the surprisingly strong (.83, standardized) association between gf and PPVT in the full sample is similar to previous research in children (Naglieri, 1981) and adults (Bell et al., 2001), although with small, typically developing samples using different statistical methods.

Supplementary Table 1. Correlation matrix for cognitive and neural measures in the CALM sample.

Supplementary Table 2. Correlation matrix for cognitive and neural measures in the NKI-Rockland sample.

Supplementary Fig. 1. Left: SEM Tree results for the relationship between gc and the anterior thalamic radiations (ATR) (see Table 3 for full results). Figure adapted from Fuhrmann et al., 2019. Right: Visualization the nature of the effect for one path (ATR->gc). The association between FA in the ATR and scores on the gc factor are moderately to strongly positive in the youngest children (r=.22) and the oldest children (r=.29), but effectively absent in the intermediate group (r=0.02). Notably, the reader can see in this figure the steady increase in fractional anisotropy across ages (scaled ATR scores moving rightward) and improvement in gc (gc scores moving upwards).


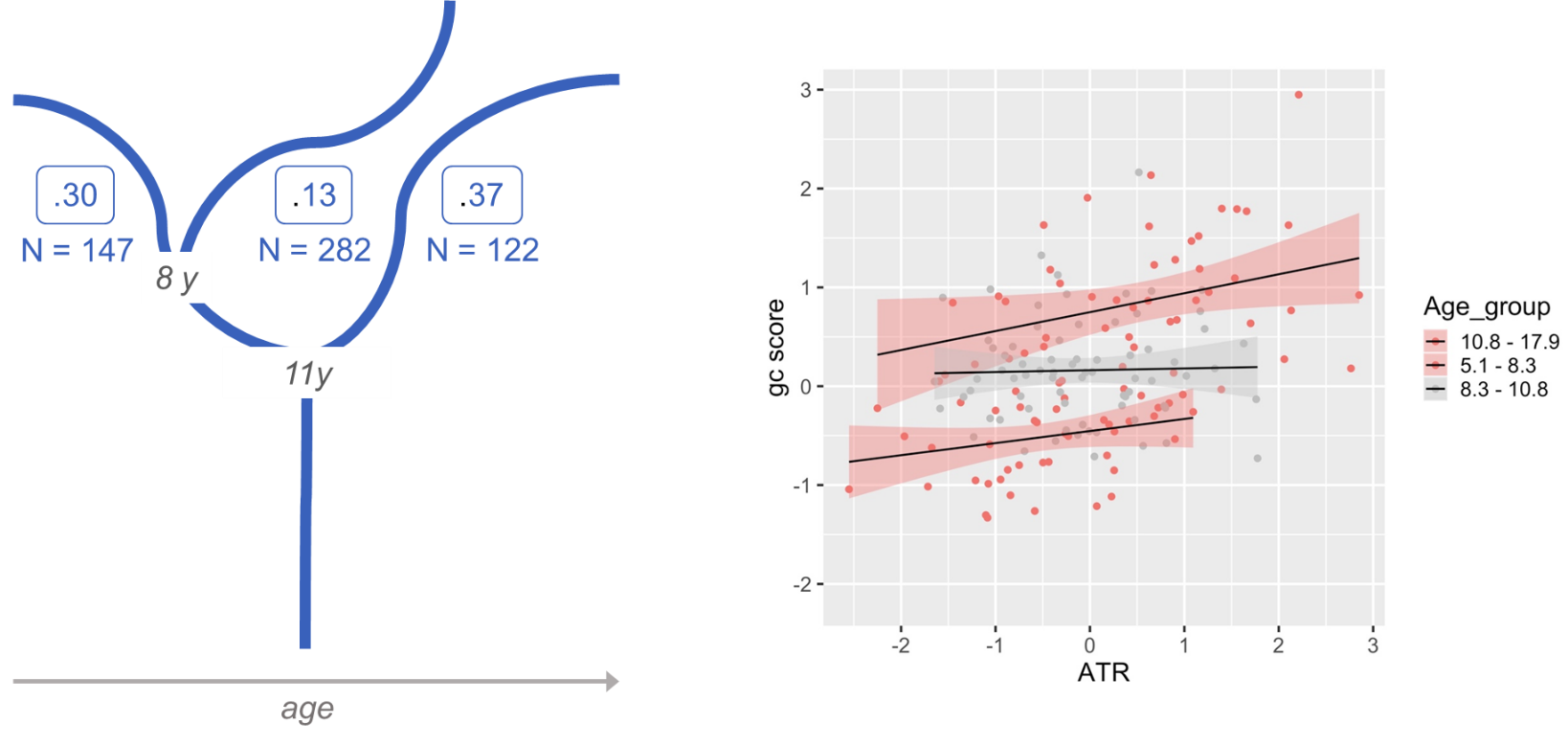


**References**

Bell, N.L., Lassiter, K.S., Matthews, T.D., Hutchinson, M.B., 2001. Comparison of the

Peabody Picture Vocabulary Test—Third Edition and Wechsler Adult Intelligence

Scale—Third Edition with university students. Journal of Clinical Psychology 57, 417–

422. <https://doi.org/10.1002/jclp.1024>.

Naglieri, J.A., 1981. Concurrent validity of the revised Peabody Picture Vocabulary Test.

Psychology in the Schools 18, 286–289. https://doi.org/10.1002/1520-

6807(198107)18:3<286::AID-PITS2310180306>3.0.CO;2-1.
